# The benefits of propofol on cancer treatment: Decipher its modulation code to immunocytes

**DOI:** 10.3389/fphar.2022.919636

**Published:** 2022-11-04

**Authors:** Long Gu, Xueqi Pan, Chongcheng Wang, Lei Wang

**Affiliations:** ^1^ First Operating Room, First Hospital of Jilin University, Changchun, China; ^2^ Intensive Care Unit, First Affiliated Hospital to Changchun University of Chinese Medicine, Changchun, China; ^3^ Trauma Center, Shandong Provincial Hospital Affiliated to Shandong First Medical University, Jinan, China; ^4^ Department of Pediatric Neurology, First Hospital of Jilin University, Jilin University, Changchun, China

**Keywords:** cancer resection, propofol, immunocytes, inflammation, immunomodulation

## Abstract

Anesthetics are essential for cancer surgery, but accumulated research have proven that some anesthetics promote the occurrence of certain cancers, leading to adverse effects in the lives of patients. Although anesthetic technology is mature, there is no golden drug selection standard for surgical cancer treatment. To afford the responsibility of human health, a more specific regimen for cancer resection is indeed necessary. Immunosuppression in oncologic surgery has an adverse influence on the outcomes of patients. The choice of anesthetic strategies influences perioperative immunity. Among anesthetics, propofol has shown positive effects on immunity. Apart from that, propofol’s anticancer effect has been generally reported, which makes it more significant in oncologic surgery. However, the immunoregulative function of propofol is not reorganized well. Herein, we have summarized the impact of propofol on different immunocytes, proposed its potential mechanism for the positive effect on cancer immunity, and offered a conceivable hypothesis on its regulation to postoperative inflammation. We conclude that the priority of propofol is high in oncologic surgery and propofol may be a promising immunomodulatory drug for tumor therapy.

## 1 Introduction

Tumor is a significant disease jeopardizing human health. According to the cancer statistics in 2022 (Siegel et al., 2022), in the United States, it has been estimated that 609,360 people will die from cancer and approximately 1,918,030 new cancer cases will be diagnosed, leading to a huge burden to families and society. For most solid cancers, surgical resection is the preferred treatment method. However, due to inevitable operative tissue lesion and the use of anesthetics, surgery is commonly accompanied by the decay of the immune system (Ogawa et al., 2000; Kim, 2017; Kim, 2018). Also, there are probably many adverse effects to bear after surgery due to the release of numerous cancer cells or the suppression of the activity of anticancer lymphocytes. All of these possibly raise the recurrence risk of tumors or cause poor prognosis for patients. Research has indicated that there are intimate connections between anesthetics and cancer occurrence, development, recurrence, and surgical prognosis (Jiao et al., 2018; Moradkhani and Karimi, 2018). The selection of anesthetic strategies can be directly related to the recovery of the immune system. Propofol, first used as an anesthetic, has shown its new pharmacological functions in cancer treatment, promising to be an effective drug in the therapy of cancer.

The underlying mechanisms of propofol’s anticancer effect have been expounded by some review articles (Jiang et al., 2018; Gao et al., 2020; Xu et al., 2020). However, despite its direct suppression of tumor cells, propofol also alters the immune system (Sanders et al., 2011; Kim, 2018), which is vital to the prognosis of cancer patients, but less elucidated. In this review, we analyzed its immunomodulation function in cancer treatment based on immunocytes-related experimental research and its preponderance in cancer resection based on the interpretation of the clinical research, revealing that propofol-mediated COX inhibition in macrophages contributed to its antitumor immunity and propofol-profited immunity recovery after surgery. We also propose a model of sterile inflammation after cancer resection, demonstrating the beneficial effect of propofol on wound healing. Also, we conclude that propofol is approximated to be a mild immunomodulatory drug that will favor tumor therapy.

## 2 Propofol: An anesthetic

Surgical resection is the primal strategy for tumor treatment, and the development of anesthetics provides the wings for surgery to attain its present achievement. Propofol is one of the most applied anesthetics in clinical practice. Early in 1987, propofol was first approved in surgery as an anesthetic inducer in the United Kingdom. Shortly afterward, because of its excellent sedative and anesthetic effect, propofol rapidly became a generally used intravenous anesthetic agent and was applied in cancer resection surgery.

### 2.1 Brief history of anesthetics

Anesthesia is the cornerstone of modern surgery. By inducing loss of consciousness, anesthetics can help patients relieve their physical and mental sufferings. The earliest anesthetics came from the extract of natural products, and the analgesic effect of this kind of crude extract was inadequate, and it was easy to cause drug poisoning.

In 1846, Dr Morton carried out the first public representation of ether for surgery, opening the history of modern anesthesiology. At the same time, it also began the dominance of ether as an anesthetic for about 100 years. Moreover, other volatiles were proposed to have analgesic effects, like nitrous oxide and chloroform.

With the consistent discovery of natural anesthetics, synthetic anesthetics have also been studied. In 1905, the local anesthetic procaine came out, which solved the shortcomings of cocaine as a local anesthetic. After that, lidocaine was also successfully invented. In the synthesis of general anesthetics, sodium thiopental, used in clinical trials in 1934, has been undoubtedly one of the most dominant general anesthetics in the 20th century. Also, halothane, which rapidly replaced ether and chloroform (Gyorfi and Kim, 2020) in 1956, could not shake its dominant position. As an inhalational anesthetic, halothane was replaced by isoflurane and sevoflurane in the 1990s.

The side effects of thiopental were gradually exposed after it being used for more than 50 years. Therefore, it was necessary to seek more safe and effective intravenous anesthetics, and propofol discovered by John Baird Glen emerged as the times require and has become a standard inducer for surgical anesthesia (Walsh, 2018). Propofol has a unique anesthetic balance and has minimal impact on respiration and heart rate. In the mouse study, It can be taken repeatedly in mice, and there is no cumulative “hangover” effect, indicating that the metabolism is fast. The pharmacokinetic and metabolic studies in humans showed that >99% of propofol was metabolized in the liver. The liver P450 enzyme can oxidize propofol to generate water-soluble, excretable metabolites which can be excreted by the kidneys. The rest is oxidized by liver P450 enzyme to generate water-soluble, excretable metabolites.

### 2.2 Mechanism of propofol’s anesthetic effect

The molecular mechanism of anesthesia is not completely clarified. The Meyer–Overton rule was the most prevalent mechanism to explain anesthesia in the 20th century (Bovill, 2000). Meyer and Overton proposed that the potency of an anesthetic was proportional to its lipid solubility, and it affected nerve function by disturbing the lipid bilayer (Perouansky, 2015). However, with the revelation of the protein complexes, the classical anesthesia theory gradually gave way to the ion channel theory. According to this, general anesthetics regulated the ion flow of nerve cells by affecting the switch of specific ion channels on the cell membrane, such as the transmitter-gated ion channels (TGICs), to achieve anesthesia (Weir et al., 2017). The TGIC can be roughly divided into two types. The inhibitory type, represented by GABAAR, is composed of five transmembrane subunits. After binding with the receptor, the ligand can cause channel opening, anion influx, and neuronal hyperpolarization. The excitatory receptor, like N-methyl-D-aspartate (NMDA), exhibited the opposite effect. Therefore, general anesthetics can enhance the inhibitory receptor or weaken the excitatory one to induce the anesthetic effect.

#### 2.2.1 Propofol is a GABAAR agonist

GABA (γ-aminobutyric acid) is the most important inhibitory neurotransmitter in the central nervous system. There are two types of GABA receptors (GABARs) (Brohan and Goudra, 2017): GABAAR and GABABR. GABABR is a slow response receptor of GABA and can inhibit the release of presynaptic neurotransmitters (Manz et al., 2019) and promote the production of inhibitory postsynaptic potential on the postsynaptic membrane by activating downstream pathways. GABAAR is a kind of ligand-gated ion channel, which responds quickly to GABA and is the main target of propofol-induced sedative effect (Brohan and Goudra, 2017). GABAAR is a chloride ion channel formed by 2 α subunits, 2 β subunits, and 1 γ subunit. Propofol binds to its extracellular part to activate the channel directly, increasing the permeability of the cell membrane to chlorine, causing chlorine influx, leading to the hyperpolarization of the cell membrane, and finally inhibiting the excitability of neurons.

#### 2.2.2 Other mechanisms for propofol to induce anesthesia

In addition to the activation of GABAAR, studies have shown that propofol can affect the opening of voltage-gated ion channels. Ratnakumari and Hemmings (1997) pointed out that propofol could induce the influx of sodium and release of glutamate by regulating the sodium channel. Protein kinase C was indicated to be involved in anesthesia of the CNS. The study by Hemmings et al. (1995)showed that propofol might induce anesthetic effect by regulating PKC phosphorylation.

## 3 Propofol: A direct cancer cell–inhibiting reagent

It has been proven that propofol displays an intimate association with the biological behavior of cancer. In some cancers, such as gallbladder cancer and breast cancer, propofol processed cancer-promoting effects, but most studies have shown its cancer-inhibiting function from not only affecting epigenetic pathways, such as those involving miRNA, lncRNA, and histone acetylation, but also modulating signaling pathways, such as the hypoxia, NF-κB, MAPK, SLUG, and Nrf2 pathways. The cancer types that could be inhibited by propofol include colon cancer (Miao et al., 2010), breast cancer (Li et al., 2012), cervical cancer (Zhang et al., 2015), glioma (Xu et al., 2018), non–small-cell lung cancer (Xing et al., 2018), cholangiocarcinoma (Zhang et al., 2019), Leydig cell cancer (Kang et al., 2019), colorectal cancer (Chen et al., 2018), thyroid cancer (Chen et al., 2019a), leukemia stem cell (Chen et al., 2020), gastric cancer (Yang et al., 2017), oral squamous cell carcinoma (Gao et al., 2019), endometrial cancer (Du et al., 2018), cardia cancer (Su et al., 2020), and so on. It can not only inhibit cancer angiogenesis, invasion, and metastasis but also reduce cancer proliferation and induce cancer cell death such as apoptosis, which has been reported by numerous articles. So, in this passage, we discuss propofol’s anticancer effect from a new direction, its immunity modulation effect, which has not been well organized by published articles.

## 4 Effect of propofol on immunocytes

Since many years, it has been proven that propofol has immunomodulation function which can regulate the resistance of the human body to cancer. Many review articles have mentioned its underlying mechanism incidentally. For instance, the effect of volatile anesthetics (sevoflurane, isoflurane, nitrous oxide, and halothane), intravenous anesthetics (propofol, ketamine, thiopental, and midazolam), and perioperative auxiliary drugs (morphine, fentanyl, sufentanil, remifentanil, alfentanil, and lidocaine) on immune function has been reviewed by Kim (2018). But due to the limited length of the article, the effect of propofol on immunity has not been well organized, and many valuable articles have been omitted. Herein, we focus on propofol’s immunomodulation function and concentrate on its effect on immunocytes, uncovering its code in cancer treatment.

**FIGURE 1:**
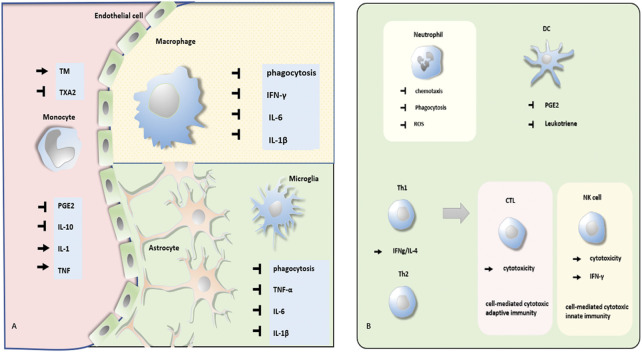
Effect of propofol on different kinds of immunocytes. **(A)** Propofol regulates phagocytosis, secretion of PGE, and cytokines of monocyte, macrophage, and microglia. **(B)** Propofol modulates the function of neutrophils, DCs, Th cells, CTL, and NK cells.

Native immunocytes are crucial for the process of innate immune response to prohibit infection, clean up tumor cells and damaged tissue, and realize homeostasis in a nonspecific manner. An adaptive immune response can powerfully sweep away unique targets. The immunomodulation of anesthetics consists of both a direct path and an indirect path. In addition to the effect on the neuroendocrine system, they can also regulate the function of immunocytes directly. As a generally used anesthetic, propofol exhibits diverse influences on multiple immune cells (Figure 1 and Table 1). In this section, the detailed depiction is as follows.

**TABLE 1:** Regulation of propofol to immunocytes.

Immunocyte	Cell resource	Effect	Mechanism	Reference
DC	Murine bone marrow	Suppresses the production of cysteinyl leukotrienes and leukotriene B4	Inhibiting 5-LO enzyme activity directly	Inada et al. (2013)
	Murine bone marrow	Suppresses prostaglandin E2 production	Inhibiting COX enzyme activity	Inada et al. (2011)
Macrophage	Mouse bone marrow	Inhibits LPS + ATP–induced pyroptosis	Suppressing activation of caspase-1 and TLR-4	Ji et al. (2020)
	RAW 264.7 and THP-1	Not attenuate IgG opsonized phagocytosis	—	Zha et al. (2019)
	RAW 264.7, RAW-ASC, and J774	Induces macrophage pyroptosis in overdose	Activating NLRP3/ASC/caspase-1 pathway	Sun et al. (2019)
	Peripheral blood and THP-1	Suppresses IL-6 and IL-1β expression in M1 macrophage	Activating GABA(A) receptor and Nrf2-mediated signaling	Kochiyama et al. (2019)
	Peripheral blood and THP-1	Reverses pressure-stimulated phagocytosis	Activating GABAA receptors and inhibiting p130cas phosphorylation	Shiratsuchi et al. (2009)
	Raw 264.7	Suppresses chemotaxis, phagocytosis, oxidative ability, and IFN-gamma production	Inhibiting mitochondrial membrane potential and ATP synthesis	Chen et al. (2003)
Microglia	BV2	Alleviates the intermittent hypoxia-induced secretion of TNF-α and IL-6	Inhibiting NF-κB and p38 MAPK signaling	Liu et al. (2017)
	Mouse brain and BV2	Inhibits the LSP induction of IL-1β from glial cells	Suppressing ERK 1/2 phosphorylation	Tanaka et al. (2013)
	HMG030	Reverses pressure-induced phagocytosis, TNF-α and nitrate production, and IL-1β secretion	—	Yu et al. (2011)
Monocyte	THP-1	Represses TNF-α–induced TM suppression	Mediating NADPH oxidase and TTP inactivation and activating HuR	Lin et al. (2011)
	Human peripheral blood	Suppresses PGE2 production and IL-10 secretion	Inhibiting COX-2 activity	Kambara et al. (2009)
	THP-1	Decreases PGE2 and TXB2 production	Inhibiting cyclooxygenase activity	Inada et al. (2009a)
	Human blood	Not affect chemotaxis	—	Krumholz et al. (1999)
	Human blood	Induces TNF and IL-1α secretion	—	Rossano et al. (1992)
NK cell	Human peripheral blood	Increases cytotoxicity of NK cells	—	Zhou et al. (2018)
	Mice spleen	Upregulates IFN-γ production	Suppressing macrophage PGE2 production	Inada et al. (2010)
	Mice spleen	Upregulates natural killer cell activity	Promoting differentiation of DCs	Inada et al. (2009b)
Neutrophil	Human peripheral blood	Inhibits superoxide generation and elastase release	Blocking formyl peptide receptor 1 (FPR1)	Yang et al. (2013)
	Human whole blood	Decreases both superoxide anion and hydrogen peroxide concentrations	—	Mühling et al. (2002)
	Human venous blood	Inhibits chemotaxis, phagocytosis, and reactive oxygen species	Decreasing intracellular calcium ion	Mikawa et al. (1998)
Lymphocyte	Human blood	Induces the secretion of interferon-γ	—	Rossano et al. (1992)
T lymphocyte	Human blood	Not cause any depression of T lymphocytes	—	Devlin et al. (1994)
T helper cell	Human peripheral venous blood	Shifts the balance between T helper cell subpopulations toward Th1-like responses	—	Salo et al. (1997)
CTL	Mice spleen	Increases the activity of CTL	—	Kushida et al. (2007)

### 4.1 Monocytes and macrophages

Leukocytes can be mainly divided into three kinds: monocytes/macrophages, granulocytes, and lymphocytes. The monocytes in the circulation come from the bone marrow and are collected into specific tissues with the effect of chemokines through the vascular endothelial cell, transforming into macrophages. Although monocytes exhibit phagocytosis and immune regulation function, macrophages are the more efficient formation. Macrophages have two kinds of polarization forms: classically activated macrophages (M1 type) and alternatively activated macrophages (M2 type). The M1-type macrophages, which are induced by LPS, IFN-γ, or TNF-α, mainly secrete pro-inflammatory factors with the consequence of tissue damage, while the M2-type macrophages activated by IL-4, IL-10, IL-13, or TGF-β produce anti-inflammatory factors, participating in wound healing.

#### 4.1.1 Monocytes

It has been reported that prostaglandin E2(PGE2) could inhibit immune function by suppressing the secretion of IL-12 or IFN-γ. While, propofol could repress, both *in vitro* and *in vivo* (Kambara et al., 2009; Inada et al., 2009a), the secretion of PGE2 from the monocytes by the inhibition of COX-2 activity, which might retard the immunosuppression effect in the postoperative phase. Besides, by directly suppressing the secretion of IL-10 (Kambara et al., 2009), an immunosuppressive cytokine, and increasing TNF and IL-1 (Rossano et al., 1992), which could increase the capability of normal T cells and activate multiple immunocytes, propofol could also promote immune function.

A study that was carried out by Krumholz et al. (1999) tested the effect of many intravenous anesthetics on the chemotaxis of human monocytes. It concluded that unlike ketamine, midazolam, and droperidol, propofol did not impair monocyte chemotaxis, which was another beneficial factor for immunity.

Propofol participates in monocytes’ regulation of postoperative coagulation reaction. Thrombomodulin (TM) and thromboxane A2 (TXA2) are two pro-coagulants. Also, Lin et al. (2011) have reported that propofol could raise the level of TM by the inactivation of NADPH oxidase and tristetraprolin (TTP) and the activation of HuR. However, another research that investigated the effect of propofol on thromboxane B2 (TXB2) production led to some inconsistencies. They found that TXB2 production (reflecting the content of TXA2) from the monocytes was decreased by suppressing the activity of cyclooxygenase in the presence of propofol (Inada et al., 2009a).

#### 4.1.2 Macrophages

Although the viability of macrophages was not affected, an appropriate concentration of propofol could regulate their function.

High-dose propofol treatment damages the immune system, possibly leading to propofol infusion syndrome (PRIS), a life-threatening complication. In 2019, to discover the molecular mechanisms, a study by Sun et al. (2019) found that propofol overdose could induce macrophage pyroptosis, a kind of programmed cell death (PCD) *via* activating the NLRP3/ASC/caspase-1 pathway. However, it has been reported that the therapeutic concentrations of propofol did not cause a decrease in macrophage viability (Chen et al., 2003). Interestingly, in 2020, a new study reported that by downregulating the expression of TLR-4 and inhibiting caspase-1 activation, LPS- and ATP-induced pyroptosis could be reversed by the administration of propofol (Ji et al., 2020).

Besides cell viability, Chen et al. (2003) found that propofol also suppressed the phagocytosis of macrophages, which was due to the inhibition of mitochondrial membrane potential and adenosine triphosphate synthesis. And another research demonstrated that propofol repressed the phagocytosis of macrophages by the activation of GABAA receptors and inhibition of p130cas phosphorylation (Shiratsuchi et al., 2009). However, research in 2019 has reported that distinct from isoflurane and sevoflurane, propofol did not abate macrophage phagocytosis mediated by opsonization—special phagocytosis enhanced by the antibody or complement bound to the target (Zha et al., 2019).

As for cytokines, propofol could inhibit INF-γ mRNA synthesis in macrophages. Also, there were some differences in polarized macrophages. Probably by regulating the GABA(A) receptor and Nrf2-mediated signal transduction, propofol prevents the release of IL-6 and IL-1β in M1 macrophages to attenuate tissue damage–related inflammatory responses, but did not affect the function of M2 macrophages (Kochiyama et al., 2019). Moreover, it has been confirmed that propofol could consistently reduce the oxidative ability of macrophages (Chen et al., 2003). Also, chemotaxis was also suppressed.

#### 4.1.3 Microglia

Microglia are special macrophages located in the brain and spine, participating in neuroinflammatory processes. Similarly, under a dose that did not affect the viability of microglial, propofol could still suppress its function (Liu et al., 2017).

It has been reported that the pretreatment of propofol could attenuate extracellular pressure–stimulated phagocytosis in human HMG030 cells (Yu et al., 2011). Also, under the treatment of propofol, the secretion of pro-inflammation cytokines was retarded (Yu et al., 2011). Through the inhibition of the NF-κB and p38 MAPK pathways, propofol alleviated the intermittent hypoxia-induced secretion of TNF-α and IL-6 (Liu et al., 2017). Also, the production of IL-1β induced by LPS could be suppressed by propofol *via* inhibiting extracellular signal-regulated kinase 1/2 (ERK 1/2) phosphorylation (Tanaka et al., 2013). Besides, the reduction of CD11b protein also reflected propofol’s inhibition of microglia.

In general, propofol may suppress the function of M1 macrophage to prevent inflammation damage but have little effect on M2.

### 4.2 Granulocytes

Due to the diversity of the nucleus, granulocytes are also named polymorphonuclear leukocytes (PMN), composed of eosinophils, neutrophils, basophils, and mast cells. Given the abundance, PMN refers in particular to neutrophils under some circumstances.

#### 4.2.1 Neutrophils

Although neutrophils are vital guardians against bacterial infection, they also exhibit tissue injury effects by producing a large number of toxic factors, and this harmful inflammation reaction could be suppressed by propofol.

It has been reported that chemotaxis, phagocytosis, and reactive oxygen species (ROS) production of neutrophils all could be blocked by propofol. Mikawa et al. (1998) examined the effect of propofol at clinically relevant concentrations and found that propofol could inhibit the function of neutrophils in a dose-dependent manner, which was supposed to contribute to the decreasing effect on [Ca2+]i in neutrophils. Another *in vitro* experiment came to the same conclusion and proved that propofol significantly decreased O^2−^ and H_2_O_2_ formation and released myeloperoxidase (MPO) (Mühling et al., 2002). Particularly, Yang et al. (2013) have shown that by the blockade of formyl peptide receptor 1 (FPR1), propofol inhibited the generation of superoxide generation and elastase in neutrophils, attenuating neutrophil-mediated inflammatory damage.

### 4.3 Lymphocytes

Lymphocytes can be generally divided into nature killer (NK) cells, T helper (Th) cells, cytotoxic T cells (CTLs), γδ T cells, and B cells. Some of these have been reported to be regulated by propofol.

#### 4.3.1 NK cells

The NK cells are a member of the innate immune system, which can recognize and destroy cancer or virus-infected cells in a nonspecific way. Dendritic cell (DC)–based vaccine injection is a special method to enhance anticancer immunity. Through experiments conducted in mice, Inada et al. (2009b) proved that propofol-differentiated DCs could significantly improve the activity of NK cells. Also, research in 2018 reported that by influencing the expression of activating or inhibitory receptors, the cytotoxicity of NK cells in postoperative patients with esophageal squamous cell carcinoma could be upregulated by propofol (Zhou et al., 2018). PGE2 could suppress the production of IFN-γ from NK cells *via* the EP4 receptor. The cytokines secreted by the NK cells, like IFN-γ, could be promoted in the presence of propofol, which might be relevant to the suppression of macrophage PGE2 production. The upregulated IFN-γ can increase the activity of macrophages, leading to increased production of IL-12 and IL-18 and resulting in further activation of NK cells (Inada et al., 2010). Also, the killing effect of NK cells on tumors would be enhanced in this way. This is consistent with the research that reported propofol as a powerful inducer of interferon-γ (Rossano et al., 1992).

#### 4.3.2 T cells

It is believed that anesthetic agents have an adverse effect on human immunity. To examine the effects of intravenous (i.v.) agents on T lymphocytes, thiopentone, methohexitone, etomidate, and propofol were administrated. Devlin et al. (1994) confirmed that among them, propofol was the only one that showed no statistically significant depression of T-cell proliferation.

According to the difference in TCR, the thymus cell can be divided into αβ T cells and γδ T cells, and αβ T cells can be subdivided into CD4+T cells and CD8+T cells, which can differentiate into T helper (Th) cells and cytotoxic T cells (CTLs). Research by Kushida et al. (2007) reported that *in vitro* activity of CTLs against EL4 (mouse lymphoma cells) was obviously greater after propofol treatment, which is probably the reason why after propofol administration, the antitumor immunity of CTLs was obviously improved.

Th1 and Th2 cells are two kinds of Th cells coming from non-differentiated Th0 cells, inducing cell-mediated immunity and humoral immunity, respectively. Salo et al. (1997) launched the research to compare the Th1/Th2 balance modulation function between thiopentone and propofol. The result illustrated that distinct from thiopentone, propofol did not suppress Th cell function, but shifted Th1/Th2 balance to induce Th1-like responses.

In general, by regulating the balance between Th1 and Th2 cells, propofol promotes the Th1-like responses, which may contribute to the activity of NK cells and TCLs, and the function of B cells may be not affected.

### 4.4 Dendritic cells

The antigen-presenting cell (APC), like a macrophage, B cell, or dendritic cell (DC), can present antigens to T cells, activating its function. Both the macrophage and the B cell can only present antigens to mature T cells, like activated or memory T cells. However, the DC is the most powerful APC, and the only cell that can activate naive T cells (Th0), which bridge innate and adaptive immunity. By processing antigens during innate immune responses to present them to Th0, DCs can initiate adaptive immunity. Beyond that, DCs can produce cytokines, leukotrienes, and prostanoids to regulate immune response as well. Research has reported that propofol suppresses prostaglandin E2 production from DCs by inhibiting the activity of COX enzyme (Inada et al., 2011), but the impact is limited to DCs, with no influence on IL-12/IL-10 production and T-cell proliferation. Leukotrienes can include cysteinyl leukotrienes (CysLTs) and leukotriene B4 (LTB4 ). CysLTs can mediate DC migration and maturation, and LTB4 can also modulate multiple immune processes, such as enhancing phagocytic and antimicrobial activities of the neutrophils and macrophages and stimulating the secretion of immunoglobulins by lymphocytes. Inada et al. (2013) found that by the direct inhibition of the 5-LO enzyme, propofol could also suppress the production of CysLTs and LTB4. Also, another article (Sethi et al., 2012) pointed out that suppression of leukotriene production with propofol could decrease metastasis after tumor surgery.

## 5 Propofol: A potential immunoregulative remedy in cancer treatment

### 5.1 Mechanism of anticancer effect of immunocytes

The human immune system has three basic functions: immune defense (for eradicating foreign pathogens), immune surveillance (for eliminating abnormal inner components), and immune homeostasis (for preventing normal inner constituents from attacking). Among them, immune surveillance is the key point to protecting organisms from tumor invasion.

**FIGURE 2:**
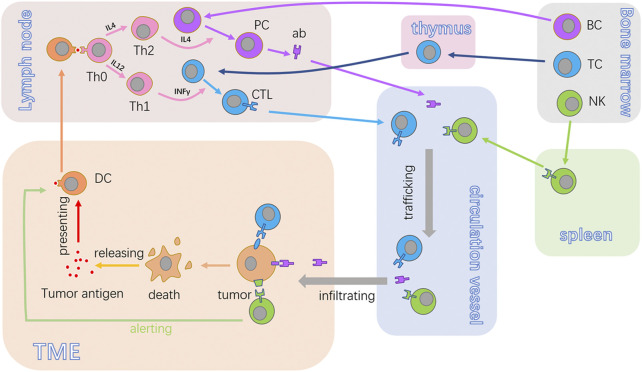
Anticancer cycle of immunocytes. BC, TC, and NK are the fundamental immunocytes in tumor immunity. The antigen released from tumor cells will be acquired by DCs. Also, DCs can present the antigen to Th0. Once activated, Th0 proliferates and differentiates into Th2 or Th1, harmonizing the activation of PC and CTL. Then, the antibodies or CTL will traffic to the TME and recognize target tumor cells and produce an effect. NK can recognize and kill cancer cells in non-special ways. BC, B lymphocytes; TC, T lymphocyte; NK, natural killer cells; ab, antibody; CTL, cytotoxic lymphocyte; PC, plasma cell; TME, tumor microenvironment; DC, dendritic cell; Th0, non-differentiated type-0 cell; Th1, type 1 T helper cell; Th2, type 2 T helper cell.

Adaptive immunity is crucial in recognizing and cleaning up the body’s tumor cells. Generally, cellular immunity is the protagonist in immune surveillance, with the assistance of humoral immunity (Figure 2). The antigen is a special substance to stimulate the body to initiate adaptive immunity. The production of adaptive immunity like antibodies and the CTL can capture its special antigen powerfully, the tension of which is known as antigenicity. Immunogenicity is the other important characteristic of antigen, which can reflect the potency to stimulate adaptive immunity. Different from antigenicity, immunogenicity is not exhibited by all antigens, and even some of them do not have the ability to initiate adaptive immunity. For those tumor antigens possessing weak or no immunogenicity, native immunity seems to undertake more obligations toward tumor prevention. These anticancer immunocytes involve NK cells, macrophages, etc.

Designating to eradicate tumor cells directly, the CTL is the essential executor of acquired immunity for cancer resistance, which can be a self-propagating process expatiated by Chen and Mellman (2013). They named this the cancer-immunity cycle, systematically illustrating the generation of cancer immunity dominated by T-cell responses (Pio et al., 2019). The closed loop consists of seven stepwise events that include the release of cancer cell antigens, cancer antigen presentation, activation of T cells, trafficking of T cells to tumors, infiltration of T cells, recognition of cancer cells by the CTL, and killing of cancer cells (Chen and Mellman, 2017). With the implementation of this cycle, immune-stimulatory factors are accumulated, amplifying the process of acquiring immunity response.

DCs coming from the bone marrow enter into the peripheral blood, distributed throughout the whole body except the brain by the blood flow (Gardner and Ruffell, 2016). Also, the DCs settling in multiple organs and nonlymphoid tissue are immature. They can highly express membrane receptors like the Fc receptor and mannose receptor, with the assistance of which DCs act out the powerful ability of antigen uptake. After the uptake, immature DCs are transformed into mature DCs, gradually, and at the same time, their migration from the peripheral tissue into secondary lymphatic organs along the lymphatics occurs.

The membrane receptors or antigen uptake, like the Fc receptor, is downregulated in mature DCs, ending in the shutting down of uptake capacity. However, their ability of antigen presentation is sharpened due to the high expression of MHC II molecules and the abundance of co-stimulatory molecules like CD80, CD86, and CD40. The existence of a co-stimulatory signal is indispensable for the activation of DC4+ naive T cells, and the DC is the only professional APC that can trigger the activation of Th0 cells.

Once the combination of tumor antigen and membrane receptors is accomplished, endocytosis of the DCs forms the endosomes, trafficking antigen protein into the lysosomes. The antigens break up into short peptides containing 10–30 amino acid residues under the efficiency of acid hydrolases.

MHC II formed in the rough endoplasmic reticulum is transferred into the Golgi complex and carried by vesicles to endosomes. After that, MHC II captures the short peptides decomposed by hydrolases, forming an antigen–MHC II complex. This complex will be transferred into the cytomembrane for antigen presentation.

With the aid of surface adhesion molecules, Th0 cells can make a temporary touch with DCs. When the TCR of Th0 recognizes the specific antigen–MHC II complex on the surface of the DC, the stable combination is achieved. Then, the CD4 of Th0 binds with the MHC II of the DC, making it more stable. Meanwhile, the relative protein of Th0 can connect with various co-stimulatory molecules, promoting the activation of Th0.

In the local microenvironment, naïve CD4^+^ Th0 cells are regulated by different cytokines to make separate differentiation. Cytokines like IL-12 and IFN-γ will hasten Th0 to realize Th1 polarization, but IL-4 can prompt it into Th2.

The cytokines produced by Th1, like IL-2 and IFN-γ, can promote cellular immunity response. The progenitor of the CTL in the secondary immune organ that captures the antigen–MHC I complex by TCR, proliferates, and differentiates into the CTL under the effect of cytokines secreted by Th1.

Guided by the chemokines, the CTL departs the lymphatic tissue for the location of the tumor. Similarly, the CTL contacts tumor cells by the adhesion molecule and recognizes them by the combination of TCR with the antigen–MHC I complex. After that, the cancer cells are executed by the CTL through the secretion of perforin/granzyme or the Fas/Fasl pathway.

The antigen–antibody immune complex can be bound to the surface of the DCs with the aid of CD21, forming iccosomes. Naïve B cells can recognize iccosomes directly depending on APC processing. Swallowed and processed by the B cells, the iccosomes combine with the MHC I and are presented to the Th cells, promoting Th2 polarization. Meanwhile, the activated Th cells express CD40L, providing the second signal for B-cell activation.

B cells shift into the plasma cells (PCs) under the influence of cytokines like IL-4, IL-5, and IL-6 secreted by the Th2 cells. The PCs are terminal B cells, most of which immigrate into the bone marrow and produce antibodies for a long time.

Although tumor antigens can induce specific antibodies and kill tumor cells by ADCC, the spontaneously produced antibody is not the key factor for tumor suppression. Some antibodies can even interfere with the immune response and promote cancer proliferation (Stambrook et al., 2017; Tokunaga et al., 2019).

The NK cell is one of the main tumor executors for cancer that lacks immunogenicity due to its nonspecific effect (Myers and Miller, 2021). The NK cells come from the bone marrow and are mainly interspersed among the peripheral blood and spleen. They can put tumor cells to death directly without the sensitization of antigens. Similar to the pattern of the cancer-immunity cycle, Bald et al. (2020) proposed the NK cell–cancer cycle to illuminate the anticancer process of NK.

The tumor microenvironment (TME) is closely related to cancer development (Wu and Dai, 2017; Arneth, 2019), consisting chiefly of different types of cells and the extracellular matrix (ECM). First, endothelial cells emerge. Tumor vessels originate from the endothelial progenitor cells, providing nutrient substances for tumor survival. The fibroblast is another participant of the TME. It has been reported to promote hematogenous metastasis of primary carcinoma. Besides, diversified immune cells, such as granulocytes, lymphocytes, and macrophages, settle in the TME. For instance, the macrophages can aggravate metastasis of tumors. The ECM is an intricate net structure by macromolecules, whose concentration can affect the intensity of tumor mobility.

The first step of the NK cell–cancer cycle is the recruitment of NK cells into the TME. Chemokines like CCL5 secreted from the immunocytes in the TME are crucial for the recruitment. Then, the NK cell membrane receptors, like the killer immunoglobulin-like receptors (KIRs) and natural cytotoxicity receptors (NCRs), recognize the tumor cells, leading to the activation of NK cells. After this, death receptor signaling or cytotoxic granules expelled by the NK cells can sentence tumor cells to death. The NK cells can also orchestrate adaptive immune responses. The cross-talk between the NK cells and DCs can bring about profound adaptive immunity to cancer.

### 5.2 Outlook of propofol in cancer suppression by immunomodulation

The influence of propofol on tumor immunology is relatively legible. Despite propofol possessing the ability to depress cancer proliferation directly, stimulating immunity of the human body is a more positive way to confront potential residual cancer cells.

**FIGURE 3:**
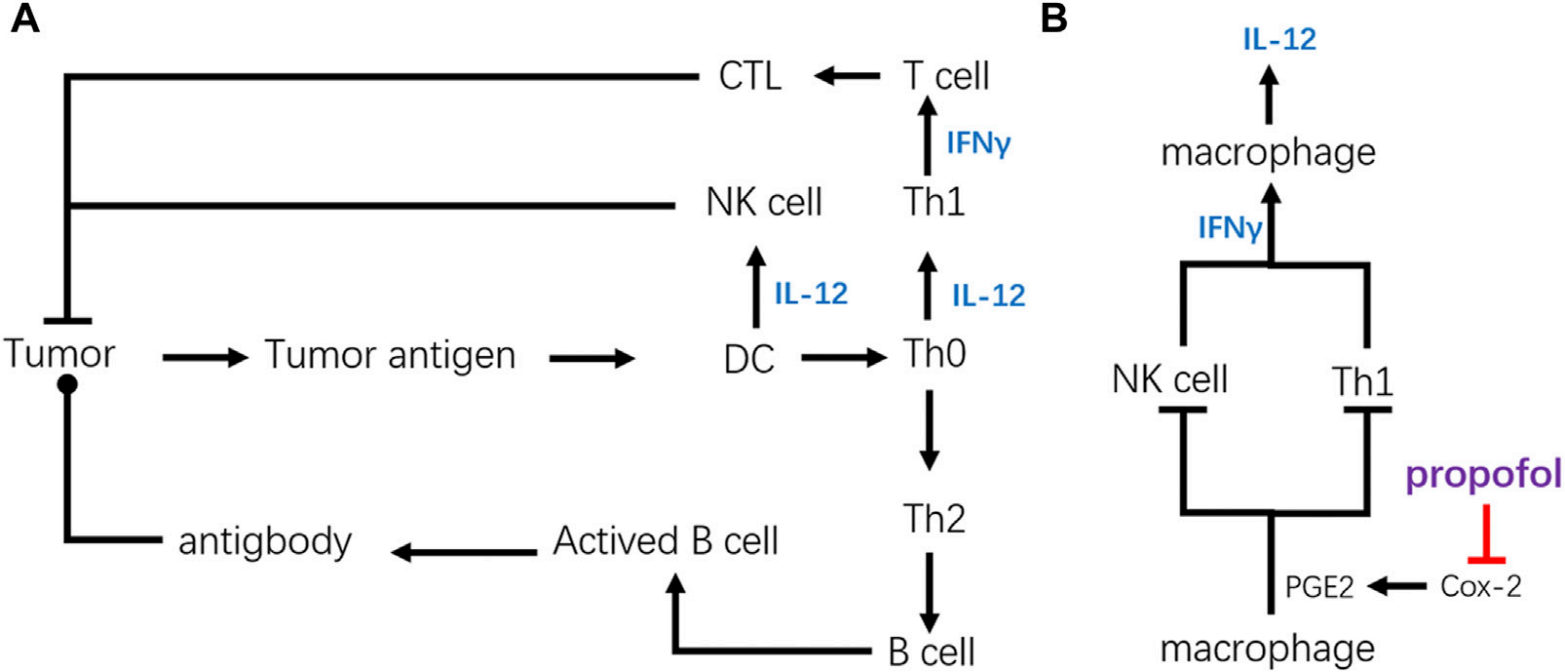
Structural diagram of tumor immunity and key regulating point of propofol. **(A)** Simplified frame of tumor immunity. **(B)** Key point of propofol to promote anticancer immunity. By inhibiting the secretion of PGE2, propofol can upregulate anticancer immunity of CTL and NK cells *via* upregulating cytokines like IFN-γ and IL-12.

The frame of tumor immune is simplified in Figure 3A. First, we discussed the effect of propofol on cellular immunity and humoral immunity. IFN-γ and TNFβ produced by the human Th1 subset can induce cell-mediated immunity. IL-4, IL-5, and IL-9 produced by the Th2 subset can promote humoral immunity. Rossano et al. found that propofol could promote unstimulated lymphocytes to produce both IL-4 and IFN-γ, while other anesthetics like ketamine or thiopentone could only upregulate the production of IL-4 but not IFN-γ. This might indicate that in the presence of propofol, both cellular immunity and humoral immunity were upregulated. Further research carried out by Salo et al. indicates that propofol could alter the Th1/Th2 balance judging by the production of IFNγ and IL-4. The increased IFN-γ/IL-4 ratio probably indicates that propofol could improve cell-mediated immunity by shifting the balance toward Th1-like responses. Given the IFNγ/IL-4 ratio and the complicated effect of antibodies on immunity, humoral immunity does not play an important role in propofol-induced cancer immunity.

The chief factor for anticancer immunity of propofol is focused on its influence on Th1 cells. It has been reported that the activity of CTL against EL4 cancer cells was significantly improved after propofol administration, which might be related to its stimulative effect on Th1 cells that could promote the activation of CTL.

As for NK cells, propofol could upregulate the cytotoxicity of NK cells against esophageal squamous cell carcinoma. This phenomenon may have a significant bearing on the DCs. The research by Inada et al. proved that the activity of NK cells could be upregulated by propofol-differentiated DCs. Besides, it has been reported that propofol could regulate the migration and maturation of DCs by affecting the level of leukotrienes. Also, the upregulated IFN-γ by propofol could also enhance cytotoxicity of the NK cells. All of these indicate that cell-mediated cytotoxic innate immunity participates in propofol-induced cancer immunity.

As shown in Figure 3A, the increased production of IL-12 is the reason for the upregulation of anticancer immunity by activating NK cells and CTLs. Cytokines like IL-12 can hasten Th0 to realize Th1 polarization, activating cellular immunity. Also, IL-12 secreted by the DCs, macrophages, or other cells can raise the activation of NK cells.

Figure 3B explains the mechanism of IL-12 accumulation, and the decrease of prostaglandin E2 (PGE2) secretion due to the COX2 inhibition is pivotal. PGE2 is important in modulating immunity, by suppressing the production of IFN-γ secreted from the NK or Th cells. Consequently, innate immunity of NK cells and adaptive immunity activated by Th1 cells could be downregulated by PGE2. Because of its ability to produce a vast amount of PGE2, the macrophage is one of the most powerful cells in the regulation of immunity. Inada et al. found that propofol could suppress the activity of COX, especially COX-2, due to the similarity of its chemical structure to a COX inhibitor, γ-tocopherol, suppressing the production of PGE2. The suppression of PGE2 production could raise the production of IL-12, which promotes the activity of CTL by promoting Th1 polarization.

E-prostanoid receptors include EP1, EP2, EP3, and EP4. By binding with EP4, PGE2 can directly inhibit IFN-γ production by NK cells (Walker and Rotondo, 2004). It has been reported that the suppression of PGE2 by propofol led to the upregulation of IFN-γ secreted by NK cells. In turn, increased IFN-γ enhanced IL-12 and IL-8 secretion by macrophages, leading to further activation of NK cells in the macrophages: NK cell co-culture. This indicates the underlying mechanism of activation of the NK cells by propofol.

Overall, the promotion of the anticancer effect of CTL and NK cells is probably due to the inhibition of COX-2 in macrophages by propofol, the descending secretion of PGE2. The decrease in PGE2 likely contributes to the upregulation of IFN-γ and IL-12, finally promoting the activities of NK cells and CTLs.

## 6 Immunomodulation function of propofol in cancer resection

Surgery is the primary selection for most cancer treatments, and in this section, we discuss the function of propofol in cancer resection based on immunity.

### 6.1 Surgery and immunity suppression

The immune system is the guardian of the organism. Its normal operation protects the human body from external threats and updates the internal components to maintain their functional conditions. The disorder of immunity, which includes both enhancement and reduction, brings troubles. Immunity can be affected by many factors, like the emotional state, nutritional status, and drug administration. Herein, we briefly demonstrate the variations of immunity in the patients undergoing surgery.

#### 6.1.1 Surgical lesion and immunosuppression

**FIGURE 4:**
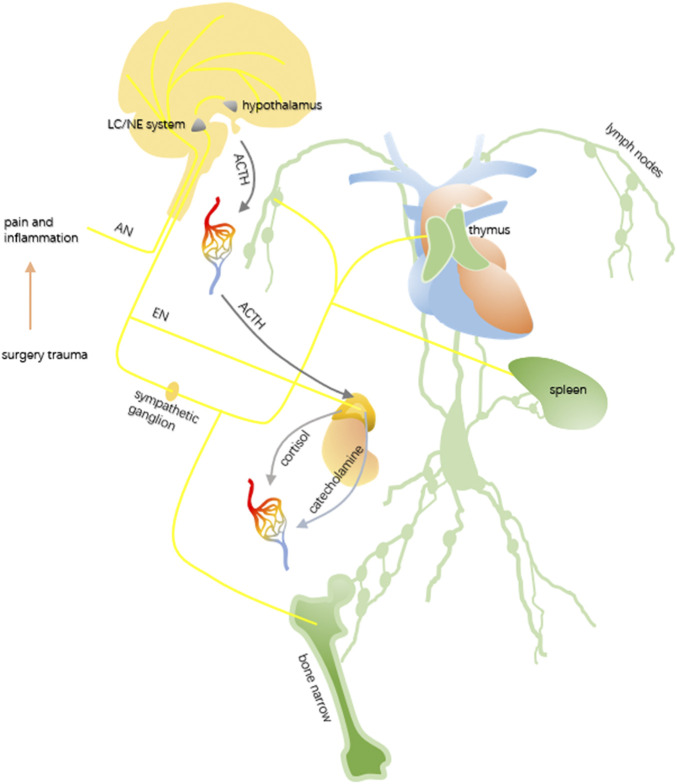
Simplified mechanism of immunosuppression induced by surgery damage. Tissue injury caused by surgery stimulates the afferent neuron (AE). The cerebrum accepts the stimulation, processes the information, and sends them to a special function unit. The locus coeruleus–noradrenergic system (LC/NE system) rapidly transmits the instruction to the immune organs and adrenal medulla through the efferent neuron (EN) and sympathetic nervous system (SNS). Affected by the hypothalamus, the anterior pituitary secretes the adrenocorticotropic hormone (ACTH) to promote the release of cortisol. Both of these contribute to the inhibition of the immune system.

Immunosuppression is a common biological response induced by surgery stress and the stress system, which includes the hypothalamic-pituitary-adrenal (HPA) axis and sympathetic nervous system (SNS), participates in the process (Charmandari et al., 2005). Without the consideration of tranquilizers and anesthetics, tissue damage under surgical operation can cause local inflammation and pain (Manou-Stathopoulou et al., 2019), both of which stimulate the sensors of the afferent nerves. After neurotransmission, the brain receives nerve impulses and commands the whole body through the HPA axis and SNS (showed in Figure 4).

Influenced by the corticotrophin-releasing hormone (CRH) coming from the paraventricular nucleus of the hypothalamus, the anterior pituitary secretes the adrenocorticotropic hormone (ACTH), prompting the adrenal cortex to release glucocorticoids (e.g., cortisol). As a lipophilic hormone, glucocorticoids can easily pass through the cell membrane and bind to the corresponding receptors, the glucocorticoid receptors (GRs), in the cytoplasm. After being activated by the glucocorticoids, the GR can translocate into the nucleus, exhibiting its genomic effects in the form of a homodimer or monomer to inhibit inflammation (Vandewalle et al., 2018).

Distinct from the parasympathetic nervous system, the SNS is crucial in the regulation of immune responses (Janig, 2014). Both central immune organs, which include the bone marrow and thymus, and peripheral immune organs, like the lymph nodes, spleen, and mucosal-associated lymphoid tissue (MALT) are innervated by the SNS, and the immune cells with adrenoceptors may be dominated by the noradrenaline released from the sympathetic postganglionic neurons, causing adrenergic stress–induced immunosuppressive effects. The locus coeruleus–noradrenergic system (LC/NE system) (Aston-Jones and Waterhouse, 2016; Benarroch, 2018), comprising the locus coeruleus and other noradrenergic cell groups in the brainstem, is another vital functional unit during stress response. In receiving stimulation from the brain, it can not only activate the HPA axis to increase the secretion of cortisol but also modulate the SNS. The spinal cord efferent fibers from the neurons in the LC/NE system end up in preganglionic sympathetic neurons, igniting the activity of the SNS. As mentioned previously, *via* acetylcholine, fibers from the preganglionic sympathetic neurons activate the sympathetic postganglionic neurons, which in turn innervate the primary and secondary lymphoid organs with norepinephrine. Besides, the preganglionic sympathetic neurons can also directly activate the adrenal medulla through acetylcholine, which is also termed the sympathetic–adrenal medulla system, to increase the secretion of catecholamines such as norepinephrine and epinephrine. Given the fact that adrenergic receptors extensively exist on the surface of the immune cells, the circulating catecholamines may also play a role in immunomodulation (Sharif et al., 2018).

Generally, despite the powerful immunosuppressive functions of the glucocorticoids, catecholamines also participate in the regulation. By the combination of rapid sympathetic nerve modulation to the immune organs and the gradual raise in circulating glucocorticoids and catecholamines secreted from the adrenal, the immunosuppression of surgery trauma is achieved.

#### 6.1.2 Perioperative period and immune suppression

The perioperative timeframe is a period of time embracing surgery, starting with the patients’ receival of operation notification and ending with their achieving the related treatment; the patient’s body generally returning to the basic level. Psychological stress is the basal challenge in the preoperative phase. Studies have demonstrated that 60%–80% of patients have anxiety due to the awareness of their surgery (Gursoy et al., 2016). Also, it has been confirmed that anxiety from the incoming uncharted surgery arouse stress responses through the HPA axis. After being sent into an operating room, patients begin the intraoperative experience until their transfer to the postanesthesia care unit (PACU) is completed. During the intraoperative period, the administration of anesthetics, analgesic agents, and muscle relaxants is essential. In addition to the traumatic damage to the body, anesthetic and analgesic agents can also produce immunosuppression (Longhini et al., 2020). The local anesthetic administration may but mitigate the HPA axis and SNS response by blocking the afferent neural transmission to alleviate the inhibition (Xu et al., 2016). The postoperative phase is a period of body recovery, which includes immune recovery. The intraoperative phase–induced immunosuppressive effect will last for several days, and the selection of different anesthetics can influence the suppressive duration.

### 6.2 Propofol’s immunoregulation function in cancer resection compared with volatile anesthetics

The combined intravenous–inhalation anesthesia is the most commonly used general anesthetic pattern in the neoplasm resection. Therefore, we focus on the immunomodulation of intravenous (i.v.) and volatile anesthetics here. The i.v. anesthetics usually exhibit immunosuppressive characteristics. Ketamine, midazolam, and droperidol have been proven to suppress the chemotaxis of monocytes, attenuating the immune function of postoperative patients (Krumholz et al., 1999), and thiopentone, methohexitone, and etomidate have been reported to reduce the function of T cells (Devlin et al., 1994), which may easily promote tumor dissemination and recurrence. Compared to these, propofol seems to have but little side impact on immunity. When it comes to volatile anesthetics, are there still advantages for propofol in immunity after cancer resection?

#### 6.2.1 Breast cancer

There are some complications in the effect of propofol and sevoflurane on immune function in patients undergoing breast cancer surgery. Some clinical researchers have reported that there are not many differences in lymphocyte function or cytokine secretion after propofol or sevoflurane treatment (Lim et al., 2018; Oh et al., 2018) (Deegan et al., 2010). Lim et al. had detected no differences in the NK cell count, CTL count, or cytokines between blood samples of the studied groups. In 2018, similarly, Oh et al. had reported that there were no intergroup differences in type 1 and type 17 T helper cells, NK cells, cytotoxic T cells, cytokines, or the neutrophil-to-lymphocyte ratio. The serum concentrations of 11 cytokines [interleukin 1β (IL-1β), IL-2, IL-4, IL-5, IL-6, IL-8, IL-10, IL-12p70, IL-13, interferon γ, and tumor necrosis factor α) and three MMPs (MMP-1, MMP-3, and MMP-9) were measured by research, and only a minority of cytokines were different between the two groups, which means these two drugs exhibit a similar function in regulating perioperative cancer immunity. Nevertheless, when compared to sevoflurane, propofol had a better impact on immunity due to its preservation for NK cell cytotoxicity (NKCC) (Cho et al., 2017). A total of 50 patients undergoing breast cancer resection were analyzed by Cho et al. to demonstrate the influence of these on the function of NK cells, and the results revealed that when compared with sevoflurane, propofol significantly increased NKCC (%). As for desflurane, while both these had a favorable impact on immunity, desflurane seemed to be the better of the two (Woo et al., 2015). Research in 2015 indicated that in terms of preservation of IL-2/IL-4 and CD4^+^/CD8+T cell ratio, both propofol and desflurane anesthesia induced a favorable immune response in the perioperative period. But when it came to leukocytes and NK cells, desflurane exhibited fewer adverse immune responses than did propofol.

#### 6.2.2 Colorectal carcinoma

Research has revealed that propofol had less influence on lymphocytes than sevoflurane (Chen et al., 2015). This research showed that the percentage of CD3⁺, CD4⁺, and CD19⁺ subtypes increased immediately after surgery, while the percentage of NK cells significantly decreased. But the proportion of the lymphocyte subtype recovered to the preoperational baseline sooner in the propofol group than it did in the sevoflurane group. CD45RO+ cells are the functional form of T cells, while CD45RA+ cells are inactive T cells. In another survey, the levels of CD45RA+ and CD45RO+ of both groups significantly decreased after surgery, while the CD45RA+ of all recovered at the postoperative 72 h, whereas the level of CD45RO+ recovered less in the sevoflurane group. According to the level of CD45RA+/CD45RO+, the postoperative immunosuppression in the treatment group of sevoflurane lasted a longer time than it did in the propofol group (Yu et al., 2019).

#### 6.2.3 Other cancers

In tongue cancer, the percentages of CD3(+) cells, CD3(+) CD4(+) cells, NK cells, and the CD4(+)/CD8(+) ratios after an operation were significantly lower in the sevoflurane groups (Zhang et al., 2014), while propofol exhibited a better effect on the recovery of immunity. The activation and differentiation of T helper cells are essential in perioperative antitumor immunity. The percentage of CD4(+)CD28(+) and ratio of interferon-gamma:interleukin-4 were evaluated in a research. Compared to isoflurane in non–small-cell lung cancer, propofol showed a more significant effect on the activation of peripheral Th cells (Ren et al., 2010). In addition, Liu et al. (2016) found that the counts of CD3^+^ cells, CD4^+^ cells, NK cells, and the CD4+/CD8+ ratios were significantly lower in the sevoflurane group, which meant that the function of the lymphocyte subsets in cervical cancer patients undergoing radical hysterectomy seemed to be better in the presence of propofol than it is with sevoflurane. However, in kidney cancer surgery, a study analyzing the amount of NK cells, total T lymphocytes, regulatory T cells, T helper cells, cytotoxic T lymphocytes, and their subpopulations concluded that there were no significant differences between propofol and sevoflurane in many kinds of lymphocytes (Efremov et al., 2020).

Generally speaking, in terms of the clinical results in many kinds of different cancer resections (showed in Table 2), it is probable that propofol possesses more benefits in postoperative immunoregulation than do sevoflurane and isoflurane, but less than desflurane. Despite that, there are still some divergences and more research is needed.

**TABLE 2:** Comparison of immunomodulation between propofol and volatile anesthetics in cancer resection.

Type of cancer resection	Anesthetics species	Comparing object	Consequence	Sample collecting time	Reference
Breast cancer surgery	Propofol and sevoflurane	Natural killer (NK) cell, cytotoxic T lymphocyte (CTL) counts, and apoptosis rate	No difference	After inducing anesthesia and at 1 and 24 h postoperatively	Lim et al. (2018)
	Propofol–ketorolac and sevoflurane–fentanyl	NK cell cytotoxicity	Propofol–ketorolac demonstrated a favorable impact on immune function	Before and 24 h after surgery	Oh et al. (2018)
	Propofol/paravertebral and sevoflurane/opioid	Concentrations of 11 cytokines and 3 MMPs	Propofol/paravertebral alters a minority of cytokines influence	Before and after surgery	Deegan et al. (2010)
	Propofol *versus* sevoflurane	Regulatory T cells, types 1 and 17 T helper cells, natural killer cells, and cytotoxic T cells	No differences	Immediately before anesthesia induction and at 24 h postoperatively	Cho et al. (2017)
	Propofol or desflurane	Lymphocyte subpopulations, concentrations of IL-2 and IL-4	Desflurane anesthesia is associated with less adverse immune responses	Before and 1 h after anesthesia induction and at 24 h postoperatively	Woo et al. (2015)
Radical resection of colorectal cancer	Propofol and sevoflurane	T lymphocyte subsets	Propofol has better impact on T lymphocyte function	Before anesthesia , 90 min after induction, 150 min after induction, and 30 min after entering post-anesthesia care unit	Chen et al. (2015)
	Propofol and sevoflurane	Lymphocyte subtype	Propofol may have less or shorter impact on immunity	Before induction, on finishing the surgery and 24 h after surgery	Yu et al. (2019)
Tongue cancer	Propofol and sevoflurane	T lymphocyte subsets, natural killer cells, and B lymphocytes	Propofol has slightly less effect on cellular immune responses	30 min before induction, 1 h, 3 h, and 5 h after induction, at the end of the operation, and 24, 48, and 72 h after operation	Zhang et al. (2014)
Pulmonary lobectomy for non–small-cell lung cancer	Propofol or isoflurane	CD4(+)CD28(+) percentage and the ratio of interferon-gamma:interleukin-4	Propofol promotes activation and differentiation of peripheral T helper cells	Before induction, 10 min after induction, immediately after stopping of anesthetics , 1 and 24 h post-operation	Ren et al. (2010)
Kidney cancer surgery	Propofol and sevoflurane	Amount of NK cells, T lymphocytes, regulatory T cells, and T-helper cells, CTL	No significant differences	Before surgery, at the end of the surgery and postoperative days 1, 3 and 7	Liu et al. (2016)
Radical hysterectomy for cervical cancer	Propofol and sevoflurane	T lymphocyte subsets and CD4+/CD8+ ratio, NK cells, and B lymphocytes	Propofol is superior in the protection of circulating lymphocytes	At 30 min before induction, the end of the operation, and 24, 48, and 72 h after operation	Efremov et al. (2020)

## 7 Propofol promotes wound healing by modulating inflammation in surgical cancer treatment

Inflammation is an ineluctable process after surgical treatment, and in spite of the immune reaction, immunocytes also participate in the process of inflammation.

### 7.1 Model of inflammation after cancer resection

Inflammation is a defense reaction of living creatures to resist harming factors. Its function is to clean up damaging cells and recover tissue injury, which include the three basic processes—alteration, exudation, and proliferation. It is an extremely delicate and intricate biological process with hundreds of thousands of uncovering mysteries. Herein, we depict the basic mode of the inflammation process and put forward the key regulating points of propofol in it. The inflammatory response is coordinated by a large range of mediators that form complex regulatory networks (Kuprash and Nedospasov, 2016). To dissect these complex networks, it is helpful to place these signals into functional categories and distinguish between inducers and mediators of inflammation. Inducers are signals that initiate the inflammatory response. They activate specialized sensors, which then elicit the production of specific sets of mediators. The mediators, in turn, alter the functional states of tissues and organs, where the effectors settle, in a way that allows them to adapt to the conditions indicated by the particular inducer of inflammation. Thus, a generic inflammatory ‘pathway’ consists of inducers, sensors, mediators, and effectors, which determines the type of inflammatory response (Medzhitov, 2008; Medzhitov, 2010).

The conserved microbial products, such as lipopolysaccharide, are referred to as pathogen-associated molecular patterns (PAMPs), and they activate pattern recognition receptors (PRRs). The PRR signaling pathways have been well characterized as the initiators of cascades that eventually lead to the migration of leukocytes to the site of infection. In spite of this, inflammation in the absence of pathogens and their products is referred to as sterile inflammation, which is induced by sterile injury like cancer resection. The immunostimulatory molecular patterns in sterile inflammation differ from microbial patterns and are canonically associated with damage (Zindel and Kubes, 2020); thus, they are called damage-associated molecular patterns (DAMPs). DAMPs are released during tissue damage and initiate an inflammatory response. Sterile inflammation and subsequent tissue repair depend on a well-orchestrated migration sequence of leukocytes to and from the site of injury.

Sensors, such as toll-like receptors (TLRs), are expressed on specialized sentinel cells, such as tissue-resident macrophages, DCs, and mast cells. They induce the production of mediators, which include cytokines, chemokines, bioactive amines, eicosanoids, and products of proteolytic cascades such as bradykinin. Mast cells are present in most tissues characteristically surrounding blood vessels and nerves and are especially prominent near the boundaries between the outside world and the internal milieu, such as the skin, mucosa of the lungs, and digestive tract, as well as the mouth, conjunctiva, and nose. Herein, we assign the conception of the sentinel cell to mastocyte and exhibit the process of sterile inflammation. The mast cell is the initiator of inflammation and plays a key role in the inflammatory process (da Silva et al., 2014). Mast cells can be stimulated to degranulate by allergens through cross-linking with immunoglobulin E receptors (e.g., FcεRI), physical injury through pattern recognition receptors for damage-associated molecular patterns (DAMPs), microbial pathogens through pattern recognition receptors for pathogen-associated molecular patterns (PAMPs), and various compounds through their associated G-protein–coupled receptors (e.g., morphine through opioid receptors) or ligand-gated ion channels (Redegeld et al., 2018). When activated, a mast cell can either selectively release (piecemeal degranulation) or rapidly release (anaphylactic degranulation) “mediators,” or compounds that induce inflammation, from storage granules into the local microenvironment (Mukai et al., 2018). In the phase of alternation, the stimulators from injuring tissue activate mast cells. Once activated, the multiple inflammatory mediators (like histamine, leukotriene, prostaglandin D2, IL-1, IL-4, IL-8, TNF, and so on) are released. Some of them (like histamine, leukotriene, IL-1, and TNF) induce the contraction of vascular endothelial cells, enlarging the endothelial cell gap, and finally increasing vascular permeability. Exudation of white cells is another feature of inflammation. Cytokines like TNF and IL-1 can increase the expression of integrin ligands in the endothelial cell, which can promote the adhesion of white cells. Under the action of chemotactic agents (like LTB4 and IL-8), white cells are attracted to the injured place through the blood vessel. The neutrophils are the most abundant leukocytes in the blood and rapidly infiltrate the target tissues. Monocytes are recruited second in the phase of exudation due to the release of the monocyte chemotactic agent from neutrophils (Kolaczkowska and Kubes, 2013). The leukocytes, mainly referring to neutrophils, recognize and degrade damaging tissue and hazard factors through phagocytosis to maintain homeostasis, and then the regeneration of the peripheral tissue realizes the healing process.

In spite of the fact that neutrophils exhibit positive aspects in tissue recovery, during phagocytosis, the leakage of lysosomal content into the extracellular stroma exacerbates tissue damage (Amulic et al., 2012). This is also a common phenomenon causing signs of swelling, pain, redness, heat, and loss of function, which makes the proper regulation of neutrophil function significant.

### 7.2 Propofol possesses beneficial influence on sterile inflammation in tissue recovery

**FIGURE 5:**
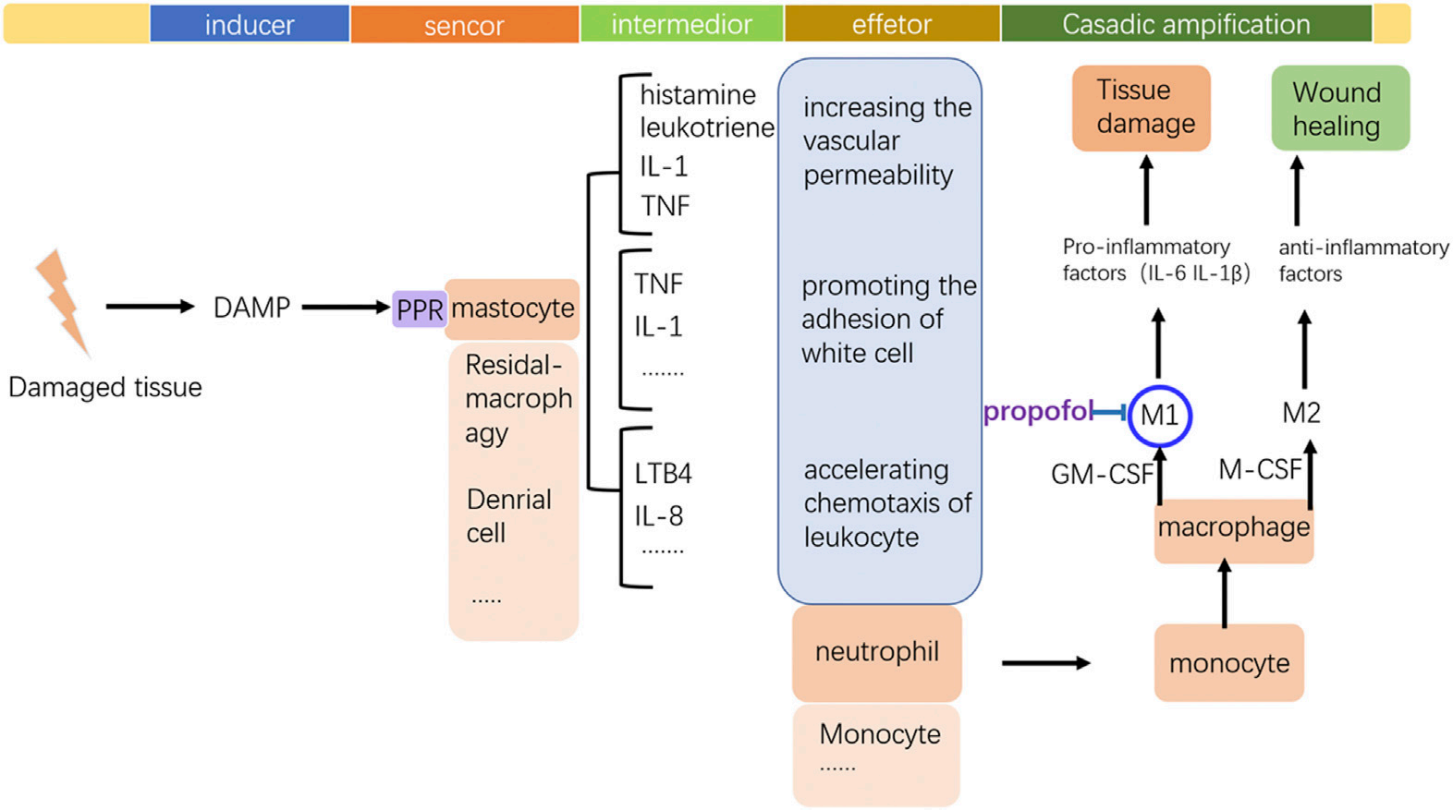
Frame of inflammation and the key modulating point of propofol to the monocyte–macrophage system. Tissue damage stimulates the occurrence of inflammation by the DAMP release. By inhibiting M1 polarization of macrophages, propofol may prevent the local tissue from excessive inflammation.

**FIGURE 6:**
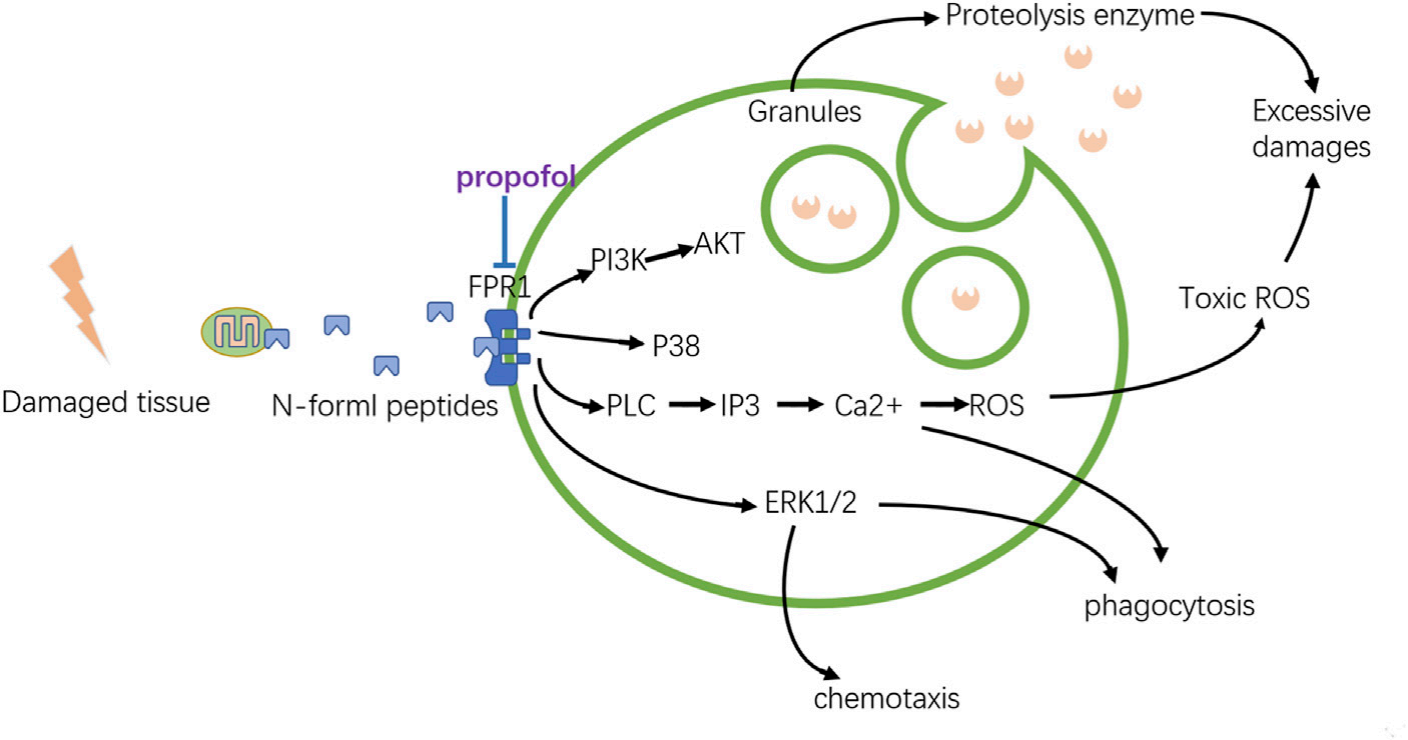
Modulation of propofol to neutrophils. The local damaged tissue can activate neutrophils by the binding of FPR1. Propofol can weaken this activation, preventing the excessive emission of proteolytic enzymes or toxic ROS, which can limit local tissue damage.

By regulating inflammation-associated immune cells, propofol plays a role in wound healing (showed in Figure 5). Although mastocytes initiate inflammation, recent research have reported that propofol could inhibit this function. Neutrophils are still the direct participants and the modality of neutrophils decides the destiny of inflammation, a favorable or detrimental one. Propofol has a direct impact on neutrophils and downregulates its function even at clinically relevant concentrations. Although neutrophils play a crucial role in the host defense mechanism as a component of nonspecific cell-mediated immunity, they are also thought to play an important role in the pathogenesis of auto tissue injury, leading to multiple organ dysfunction (Liew and Kubes, 2019). Overproduction of reactive oxygen species (ROS) by neutrophils that accumulate in the organs in response to chemotactic factors in the surrounding milieu contributes to this process. Thus, the immune function of neutrophils is a double-edged sword, and how to properly regulate its capability and prevent its overactivation is crucial. The suppression of neutrophil functions by anesthetics may be favorable to attenuate organ dysfunction mediated by the mechanism of auto tissue injury. Propofol inhibits the chemotaxis, a movement toward certain chemicals, of neutrophils, which probably attenuates its aggregation in damaged tissue. Also, phagocytosis of neutrophils is also inhibited by propofol; similar to chemotaxis, the underlying mechanism of propofols’ suppression of phagocytosis is unidentified. The decreasing effect on the intracellular calcium ion by propofol might result in depression of chemotaxis, phagocytosis, and ROS production. Delightfully, further research on propofols’ inhibition of the production of ROS and released proteolysis enzymes might imply its positive effect on inflammation (showed in Figure 6). The formyl peptide receptor (FPR) belongs to a class of G protein–coupled receptors and the class of receptors possessing seven hydrophobic transmembrane domains located in the cellular membrane of neutrophils. Human neutrophils express two members of this family: FPR1 and FPR2. Through the selective and competitive binding affinity to FPR1, propofol achieves its influence on neutrophils.

FPR1 is activated by N-formyl peptides, which can be derived from mitochondrial proteins. By binding to FPR1, N-formyl peptides released from damaged tissue can activate neutrophils and induce severe inflammatory responses. Once overwhelmingly activated, neutrophils are transformed into destructive cells, releasing toxic ROS and proteolytic enzymes that destroy the surrounding tissue. It has been proven that propofol can reduce the overproduction of ROS by inhibiting the Ca^2+^ and MAPK signaling pathways. After binding with ligands, FPR1 can activate phospholipase C (PLC), which catalyzes the conversion of phosphoinositol 4,5-biphosphate to inositol 1,4,5-triphosphate (IP3) to cause the rapid release of Ca^2+^. Besides, the ERK 1/2 MAPK cascades are also activated by FPR1, increasing the production of ROS. Both of these can just be suppressed by propofol. But how propofol represses the elastase, a major serine protease secreted by stimulated human neutrophils, released from the granules into their surroundings is still elusive. Also, the roles of PI3K/AKT signaling and p38 MAPK cascades inhibited by propofol *via* FPR1 still need further consideration.

Neutrophils are the executors of inflammation in their early phase, directly impacting the fate of inflammation, favorable or destructive. Also, the macrophage, recruited by the neutrophil to the damaging place, is probably the switch deciding the fate. During inflammatory processes, monocyte-derived (M0) macrophages undergo polarization to classically (M1) and alternatively (M2) activate macrophages, depending on the local tissue environment. Two cytokines, namely, macrophage colony-stimulating factor (M-CSF) and granulocyte M-CSF (GM-CSF), are important for priming monocyte to macrophage differentiation. M-CSF and GM-CSF stimulate monocytes to give rise to phenotypically different subsets of macrophages. M-CSF has been shown to stimulate monocyte differentiation to an anti-inflammatory, immunosuppressive macrophage phenotype (M2), while GM-CSF has been shown to stimulate a pro-inflammatory macrophage phenotype (M1). M1 macrophages are the dominating phenotype observed in the early stages of inflammation and are activated by mediators like interferon-γ (IFN-γ), tumor necrosis factor (TNF), and damage-associated molecular patterns (DAMPs). These mediator molecules create a pro-inflammatory response that in return produces pro-inflammatory cytokines. M1 macrophages recruited during the early phases of inflammation promote the production of interleukin-6, IL-1β, and tumor necrosis factor α (TNF-α), exacerbating inflammation and contributing to tissue destruction. By contrast, M2 macrophages are characterized by their involvement in immune regulation and homeostatic functions associated with wound healing. Anti-inflammatory cytokines such as interleukin-4 (IL-4) and interleukin-13 (IL-13) stimulate M2 macrophage polarization and the M2 “repair” designation (also referred to as alternatively activated macrophages) broadly refers to macrophages that function in constructive processes like wound healing and tissue repair and those that turn off damaging immune system activation by producing anti-inflammatory cytokines like IL-10. So the perioperative management to control macrophage differentiation is the point, and propofol is exactly involved in this regulation. Inhibition of IL-6 production may suppress systemic inflammation induced by surgical trauma, thereby reducing postoperative complications. It has been reported that propofol suppresses IL-6 and IL-1β expressions during human M1 macrophage polarization, suggesting that propofol plays a protective role in the development and progression of inflammation. Also, the activity of M2 macrophage is not affected by propofol.

## Conclusion

Postoperative immunosuppression is a common phenomenon and has a disadvantageous impact on patient prognosis of surgical cancer resection. Propofol, a generally used anesthetic in oncologic surgery, has been proven to have beneficial effects on immune recovery. Despite the fact that propofol cannot reverse surgery-induced immune suppression related to the activation of HPA and SNS, it can directly regulate the function of immunocytes. Although the inhibition of innate immune cells, like macrophages and neutrophils, has been generally reported, propofol can improve anticancer immunity by activating the function of lymphocytes like NK cells and TCLs. The enhancement of the cytotoxic effect of these immunocytes may be beneficial to oncologic surgery. The repression of the immune system induced by cancer surgery can facilitate the dissemination of different kinds of cancer cells (Chen et al., 2019b), which leaves a hidden trouble for cancer patients. The elevation of cell-mediated immunity by propofol is likely to make sense in this condition. In addition, multiple articles have reported the anticancer effect of propofol. The comparison with other anesthetics concludes that the priority of propofol is high in cancer surgery for its relatively less adverse impact on immunity. Moreover, propofol is hopefully to be a favorable immunoregulatory agent for cancer treatment and a potential drug for wound healing by the regulation of sterile inflammation.

However, the limitation of propofol in clinical application still exists. The commonly used anesthetics clinically include volatile anesthetics, intravenous anesthetics, and perioperative auxiliary drugs. Each type of drug has unique effects on immunity. Although we recommend propofol in cancer resection, combination administration is common in clinical practice. What kind of combinations of anesthetics and adjuvant drugs is the best for propofol in oncological surgery? Also, are there other intravenous anesthetics that possess better effects after an improved combination? Then, the existing articles mainly focus on breast and colorectal cancer, but the research on other tumors is relatively insufficient. To clarify the effect of propofol on immunity, studies on other major cancer types are needed. Finally, in spite of neutrophils, studies of propofol on immunocytes rarely reach the molecular level. More meticulous basic studies are still essential for more precise demonstration.
